# Novel Strategy to Assess the Neurotoxicity of Organic Solvents Such as Glycol Ethers: Protocol for Combining In Vitro and In Silico Methods With Human-Controlled Exposure Experiments

**DOI:** 10.2196/50300

**Published:** 2024-01-18

**Authors:** Nancy B Hopf, Laura Suter-Dick, Jörg Huwyler, Myriam Borgatta, Lucie Hegg, David Pamies, Hélène Paschoud, Ramya Deepthi Puligilla, Elena Reale, Sophie Werner, Marie-Gabrielle Zurich

**Affiliations:** 1 Center for Primary Care and Public Health (Unisanté) University of Lausanne Lausanne Switzerland; 2 Swiss Centre for Applied Human Toxicology (SCAHT) Basel Switzerland; 3 School of Life Sciences University of Applied Sciences and Arts Northwestern Switzerland Muttenz Switzerland; 4 Division of Pharmaceutical Technology Department of Pharmaceutical Sciences University of Basel Basel Switzerland; 5 Department of Biomedical Sciences University of Lausanne Lausanne Switzerland

**Keywords:** organic solvent exposure, workers, general population, neurotoxicity, blood-brain barrier, liver toxicity, human cell cultures

## Abstract

**Background:**

Chemicals are not required to be tested systematically for their neurotoxic potency, although they may contribute to the development of several neurological diseases. The absence of systematic testing may be partially explained by the current Organisation for Economic Co-operation and Development (OECD) Test Guidelines, which rely on animal experiments that are expensive, laborious, and ethically debatable. Therefore, it is important to understand the risks to exposed workers and the general population exposed to domestic products. In this study, we propose a strategy to test the neurotoxicity of solvents using the commonly used glycol ethers as a case study.

**Objective:**

This study aims to provide a strategy that can be used by regulatory agencies and industries to rank solvents according to their neurotoxicity and demonstrate the use of toxicokinetic modeling to predict air concentrations of solvents that are below the no observed adverse effect concentrations (NOAECs) for human neurotoxicity determined in in vitro assays.

**Methods:**

The proposed strategy focuses on a complex 3D in vitro brain model (BrainSpheres) derived from human-induced pluripotent stem cells (hiPSCs). This model is accompanied by in vivo, in vitro, and in silico models for the blood-brain barrier (BBB) and in vitro models for liver metabolism. The data are integrated into a toxicokinetic model. Internal concentrations predicted using this toxicokinetic model are compared with the results from in vivo human-controlled exposure experiments for model validation. The toxicokinetic model is then used in reverse dosimetry to predict air concentrations, leading to brain concentrations lower than the NOAECs determined in the hiPSC-derived 3D brain model. These predictions will contribute to the protection of exposed workers and the general population with domestic exposures.

**Results:**

The Swiss Centre for Applied Human Toxicology funded the project, commencing in January 2021. The Human Ethics Committee approval was obtained on November 16, 2022. Zebrafish experiments and in vitro methods started in February 2021, whereas recruitment of human volunteers started in 2022 after the COVID-19 pandemic–related restrictions were lifted. We anticipate that we will be able to provide a neurotoxicity testing strategy by 2026 and predicted air concentrations for 6 commonly used propylene glycol ethers based on toxicokinetic models incorporating liver metabolism, BBB leakage parameters, and brain toxicity.

**Conclusions:**

This study will be of great interest to regulatory agencies and chemical industries needing and seeking novel solutions to develop human chemical risk assessments. It will contribute to protecting human health from the deleterious effects of environmental chemicals.

**International Registered Report Identifier (IRRID):**

DERR1-10.2196/50300

## Introduction

**Figure 1:**
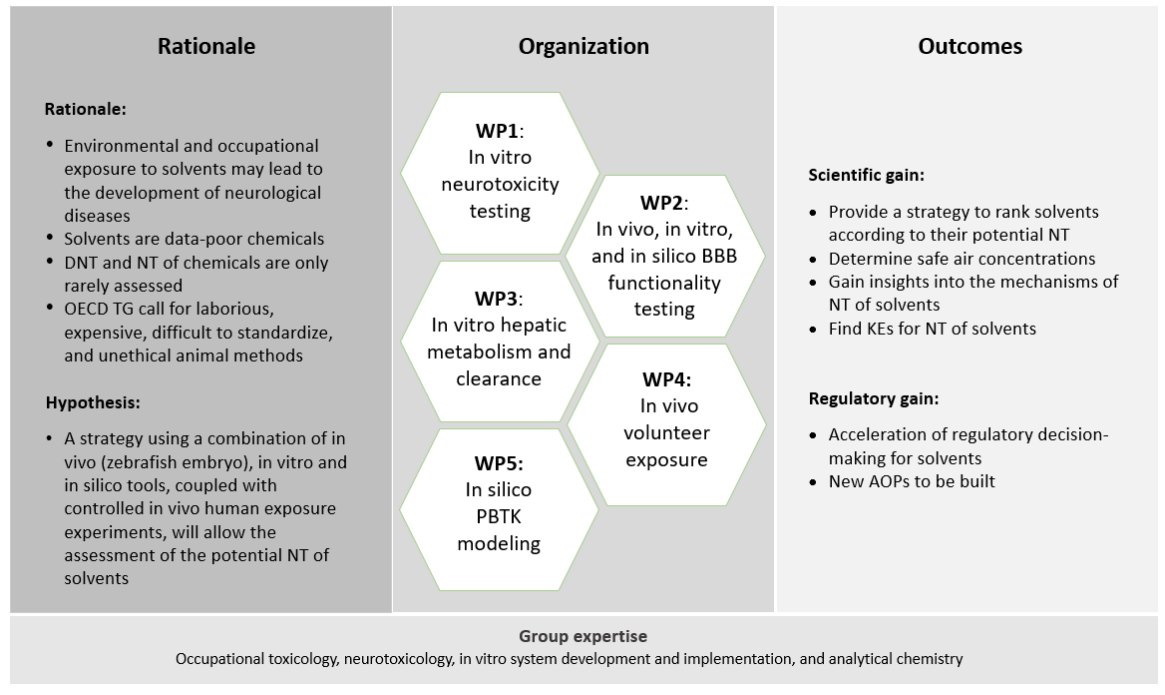
Overview of project rationale, organization, and outcomes. The project will develop a strategy based on various models to assess the neurotoxicity of solvents. Scientific and regulatory outcomes are foreseen. AOP: adverse outcome pathway; BBB: blood-brain barrier; DNT: developmental neurotoxicity; KE: key event; NT: neurotoxicity; OECD: Organisation for Economic Co-operation and Development; PBTK: physiologically based toxicokinetic; TG: Test Guideline; WP: work package.

Environmental and occupational exposure to chemicals may contribute to the development of several neurological diseases [1,2]. In particular, organic solvents used in industries such as car repair, painting, furniture manufacturing, printing, and cleaning have been associated with several central nervous system (CNS) conditions. These include mild to severe toxic encephalopathy [3]; deficits in cognitive function [4-7]; and, in some cases, neurodegenerative diseases [8,9]. The diffuse neuropathological effects of acute solvent intoxication reflect neurophysiological abnormalities involving multiple brain regions. With increasingly intense or prolonged exposure, the severity of acute impairment may progress along the spectrum of delirium. Chronic high-level exposure may lead to global cognitive impairment including deficits in memory, attention, energy, and personality, which are well-described forms of dementia [10-13]. Much of the initial work on organic solvent toxicity originated in Scandinavia, where a neurobehavioral syndrome in painters leading to their early retirement was first described [14]. However, although the neurotoxicity of solvents such as toluene, trichloroethylene, and n-hexane has been recognized, the neurotoxicity of common solvents currently on the market has not been evaluated. Notably, neurotoxicity testing is only required if the chemical is deemed a pesticide; otherwise, all other chemicals are evaluated on a case-by-case basis. The only exception is if the compound structure is suspected to have nervous system targets and no data are available for read-across or when effects on the nervous system are found in single-dose (Organisation for Economic Co-operation and Development [OECD] Test Guidelines [TGs] 402, 403, 420, 423, or 425) or repeated-dose toxicity studies (TG 407 or 408). Because the nervous system effect endpoints considered as triggers (ie, modifications of wet brain weight or basic histopathology, or both) are quite insensitive, high amounts of potentially neurotoxic compounds are available on the market. The European Union Classification, Labelling, and Packaging Regulation does not include a classification for neurotoxicity. Exposure to organic solvents, especially among workers with higher exposure than the general population, can produce neurotoxic effects, depending on the internal dose. Therefore, to efficiently protect the population from possible solvent toxicity, it is important to determine the air concentrations at which neurotoxicity does not occur. To this end, we propose a strategy using a combination of in vivo (zebrafish embryo), in vitro, and in silico tools coupled with controlled in vivo human exposure experiments to assess the neurotoxicity of solvents (Figure 1).

The recognized method for the evaluation of the neurotoxic potential of chemicals, the OECD TG 424 (neurotoxicity in rodents), uses complex in vivo tests in rodents, which are laborious, expensive, difficult to apply in a standardized manner, and ethically debatable. Regulators from different agencies worldwide as well as the scientific community are becoming increasingly aware of the limitations of the current toxicity testing paradigm. Animal-based high-dose testing in typically 1 stand-alone guideline test is not always relevant for human exposure scenarios [15]. One of the most challenging aspects of this animal-centric approach is the impossibility of coping with the thousands of chemicals for which data are still lacking. Conducting animal tests is time consuming and expensive. Therefore, they cannot be carried out routinely because of the sheer number of chemicals that are currently on the market and those anticipated to enter it in the coming years [16]. In addition, there are shortcomings regarding interspecies concordance between different mammalian or rodent species as well as with respect to extrapolation from experimental animals to humans. These ambiguities in results or poor reproducibility performance call into question the relevance of such test methods for human risk assessment [16-21]. All this prompts a move away from animal testing toward a combination of in vitro and in silico approaches that address functional mechanistic endpoints [15,16,22].

Numerous in vitro models have been proposed for the evaluation of neurotoxicity in the last decades. Monolayer cultures of a single brain cell type are far from representing the human brain in terms of architecture and functionality. Given the sophistication of brain cell-to-cell interactions, some complexity is required to recapitulate human-relevant cellular processes and functions in vitro. However, a good balance must be found between this complexity and the simplicity needed to have robust and reproducible systems that can be applied for chemical screening in a high-throughput manner [23]. The 3D hiPSC–derived brain test system (BrainSpheres) we previously developed [24] will be used in this study, as it fulfills these requirements.

The blood-brain barrier (BBB) protects the brain parenchymal cells from the deleterious effects of xenobiotics. However, some chemicals are able to cross or impair the BBB [25]. The transient or permanent opening of the BBB provides xenobiotics, plasma proteins, and immunoregulatory mediators access to the CNS, where they can induce toxic effects. Therefore, we will implement a predictive model to assess the impact of solvents on BBB based on in vivo (zebrafish embryo), in vitro (human brain microvascular endothelial cells [hCMEC/D3]), and in silico models [26] to assist in the interpretation of the results obtained in in vitro neurotoxicity testing.

Glycol ethers will be used as a case study to evaluate the feasibility of our protocol. Glycol ethers form a wide family of a few dozen solvents with different physicochemical properties making them versatile and usable in a variety of industrial applications ranging from pharmaceuticals and microelectronics to domestic cleaning, personal care, and printing. The 2 main groups of glycol ethers are the E series (ie, ethylene glycol ethers [EGEs]) and the P series (ie, propylene glycol ethers [PGEs]). EGEs and PGEs show differences in their toxicological properties regarding teratogenicity, hemolysis, and testicular atrophy [23], apparently resulting from their distinct production of metabolites [24,25]. EGEs have a primary alcohol group and are oxidized by alcohol dehydrogenase and aldehyde dehydrogenase to form the toxic alkoxyacetic acid. Therefore, PGEs are progressively introduced as a less toxic replacement of EGEs. PGEs are sold as a mixture of 2 isomers, with the bulk having a secondary alcohol (a) group and generally <5% of primary alcohol (b) groups [22]. The b-isomer is oxidized by alcohol dehydrogenase and aldehyde dehydrogenase to form the toxic alkoxypropionic acid in the body. However, the actual toxicity of PGEs is poorly characterized. Therefore, it is essential for our strategy to study not only the parent compound but also the metabolites. Metabolically competent liver cell models will be used to screen for liver toxicity of solvents and for the production of potential metabolites.

Exposure to solvents in human volunteers is fundamental when quantifying possible risks from chemicals because toxicological effects are related to internal exposure, that is, the concentration of a chemical inside the body and its biotransformation. Therefore, in vivo human volunteer studies are necessary to quantify absorption, distribution, metabolism, and excretion (ADME) kinetics in humans under controlled exposure conditions, which are necessary to understand the relationship between solvent concentrations in the air and biological samples, such as blood, urine, or exhaled air. These experiments provide important data on the total absorbed dose after inhaled solvent concentration, absorption rate (ie, from the site of absorption into the bloodstream), biotransformation rate (ie, metabolization of the parent chemical with production of metabolites), and elimination rate (ie, excretion of the parent chemical and metabolites from the body) [27]. These toxicokinetic parameters are representative of the target organ or of the tissue concentrations that may trigger an effect and are, therefore, relevant for understanding chemical risks in humans.

Toxicokinetic models quantitatively describe the body’s ADME of a chemical or substance (different terms for this concept are preferred in different fields, including “toxicokinetics,” “pharmacokinetics,” and “biokinetics”) [28]. Furthermore, using a toxicokinetic model in reverse dosimetry, we can predict the solvent air concentrations leading to brain concentrations below the levels found to produce neurotoxic effects in vitro in the BrainSpheres. The toxicokinetic model will incorporate metabolism parameters derived from the in vitro liver system and passage through or toxicity to BBB. Once calibrated, the toxicokinetic model can be used to simulate chronic exposure scenarios to predict cumulative brain concentrations and used in reverse dosimetry to predict air concentrations that will not likely result in brain concentrations associated with toxicity.

## Methods

### Ethical Considerations

Ethics committee approval was obtained from Swiss ethics (Commission cantonale d’éthique de la recherche sur l’être humain) in 2022 for this nonclinical human study (2022-01567). Healthy women and men were recruited as participants in our study. Each participant signed a written informed consent form before inclusion in the study. The participants will be reimbursed for their time and inconvenience according to the Swiss guidelines.

### Global Strategy

The choice of solvents to be included in the study will be based on the amount annually placed on the European market and the number of products registered containing known glycol ethers. The selected organic solvents will be applied to various in vitro models to determine their neurotoxicity. They must be amphiphilic to be solubilized in the cell culture media. We established the following solvent selection criteria: (1) used or produced >1 metric ton per year, (2) incorporated in numerous industrial and commercial products, and (3) water solubility. This selection process involves consulting government databases and contacting different industry sectors. We will start the project concomitantly by testing 2 solvents of the P series, propylene glycol methyl ether (PGME), for which we have already developed a toxicokinetic model, and propylene glycol butyl ether. We will test 1 additional solvent from the E series with the in vitro test system, namely, ethylene glycol methyl ether (EGME), which has been banned for use in cosmetic products in Europe [29].

**Figure 2:**
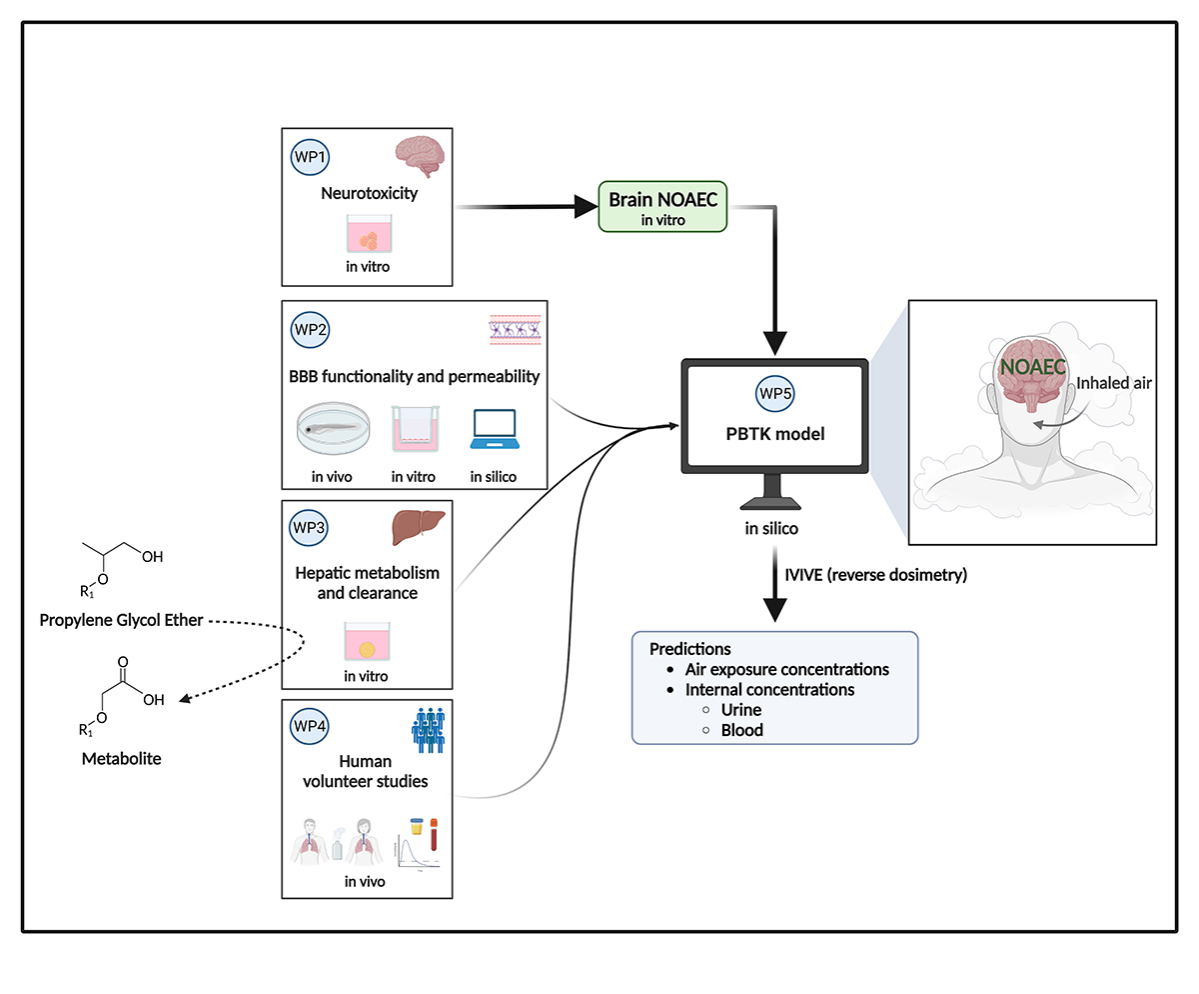
Information workflow between the work package (WP). BBB: blood-brain barrier; IVIVE: in vitro-in vivo extrapolation; NOAEC: no observed adverse effect concentration; PBTK: physiologically based toxicokinetic.

The study is organized into 5 work packages (WPs). The information workflow between the WPs is shown in Figure 2. All results collected from the abovementioned systems will contribute to refining the toxicokinetic model we previously developed for PGME [30]. The toxicokinetic parameters of the solvent and the metabolites will then be characterized in human volunteers after exposure to PGE vapors under controlled conditions. These results will be used to calibrate and expand our toxicokinetic model [30]. The toxicokinetic model will be constructed to predict brain concentrations of selected solvents and, consequently, will include a brain compartment to predict the target organ solvent and metabolite concentrations. BBB parameters such as barrier transport, transport of the solvent once in the brain, and solvent-brain binding will be incorporated. Solvent neurotoxicity may depend on the metabolic modifications of the substances; therefore, we will incorporate the parameters for the parent compound and the metabolites found in the in vitro liver system. The data necessary to build the model will be retrieved from peer-reviewed scientific literature for tissue:blood partition coefficients (PCs) [31] following the fit-for-purpose dose-response analysis approach. The toxicokinetic model should be able to predict human brain concentration for each of the tested solvents after inhalation exposure, given the air concentration of vapors and duration of exposure. The simulated human brain concentrations will then be compared with the no observed adverse effect concentrations (NOAECs) obtained from the neurotoxicity in vitro system (BrainSpheres). In addition, the toxicokinetic model will be used to predict solvent air concentrations that are unlikely to lead to brain concentration equal to or superior to the brain NOAECs using reverse dosimetry. The specific aims of each WP are summarized in Table 1.

**Table 1:** Specific aims of the work packages (WPs).

WP	Name	Specific aims
WP1	In vitro neurotoxicity testing	Determine NOAEC^a^ for neurotoxicity of each solvent Determine the in vitro distribution kinetics of solvents Identify toxicity pathways and KEs^b^ for solvent neurotoxicity
WP2	In vivo or in vitro or in silico BBB^c^ functionality testing	Evaluate the suitability of the zebrafish embryo model to study BBB integrity and functionality Determine the impact of solvents on BBB integrity and transport in zebrafish and hCMEC/D3^d^ Determine the solvents permeability coefficient (Pe) Provide quantitative data on BBB permeability and tissue distribution of solvents based on computational modeling
WP3	In vitro hepatic metabolism and clearance	Elucidate hepatic metabolism Calculate substrate-enzymatic parameters (Vmax^e^ and Km^f^) Detect and identify possible metabolites produced by the liver
WP4	In vivo volunteer exposure	Characterize human blood absorption and urinary elimination kinetics for parent glycol ether as well as the metabolites identified in WP3 Find neurotoxic and vascular injury effect biomarkers for solvent exposure
WP5	In silico PBTK^g^ modeling	Establish and calibrate the PBTK model for various organic solvents Use reverse dosimetry to determine air concentrations below human brain toxicity concentrations

^a^NOAEC: no observed adverse effect concentration.

^b^KE: key event.

^c^BBB: blood-brain barrier.

^d^hCMEC/D3: human brain microvascular endothelial cells.

^e^V_max_: maximum velocity.

^f^Km: Michaelis constant.

^g^PBTK: physiologically based toxicokinetic.

### WP1: In Vitro Neurotoxicity Testing

Testing strategies are needed to evaluate the neurotoxicity of chemicals in a more cost-effective, efficient, and ethical manner. Participating in an international effort, we developed a 3D human-induced pluripotent stem cells (hiPSC)–derived brain model containing several subtypes of neurons, astrocytes, and oligodendrocytes [24]. This system allows the cells to reach a high level of differentiation and cellular maturation, exemplified by the presence of functional synapses and compact myelin. The presence of myelin is important for this project because solvents more easily target lipid-rich structures [32]. This 3D human brain model has already proven its usefulness for neurotoxicity testing [33-36]. We hypothesized that glycol ethers are neurotoxic. Therefore, we propose to take advantage of our hiPSC-derived BrainSpheres model to study the neurotoxicity induced by uncharacterized glycol ethers present on the market, which will be compared with the neurotoxicity of well-characterized solvents known to induce human encephalopathy.

Solvents are data-poor substances. It was originally hypothesized that they exert their toxic effects largely through nonspecific physicochemical effects that modulate membrane fluidity and perturb the hydrophobic force regulating macromolecular interactions [37]. However, recent evidence supports the view that solvents interact with lipophilic areas on protein receptors [38,39]. They have also been shown to induce lipid peroxidation, leading to mitochondrial dysfunction, failure of electron transport, and energy production [40,41]. In this study, omics (eg, proteomics, metabolomics, and lipidomics) technology will be used to decipher the mechanisms of glycol ether neurotoxicity and to identify potential biomarkers of toxic effects.

**Figure 3:**
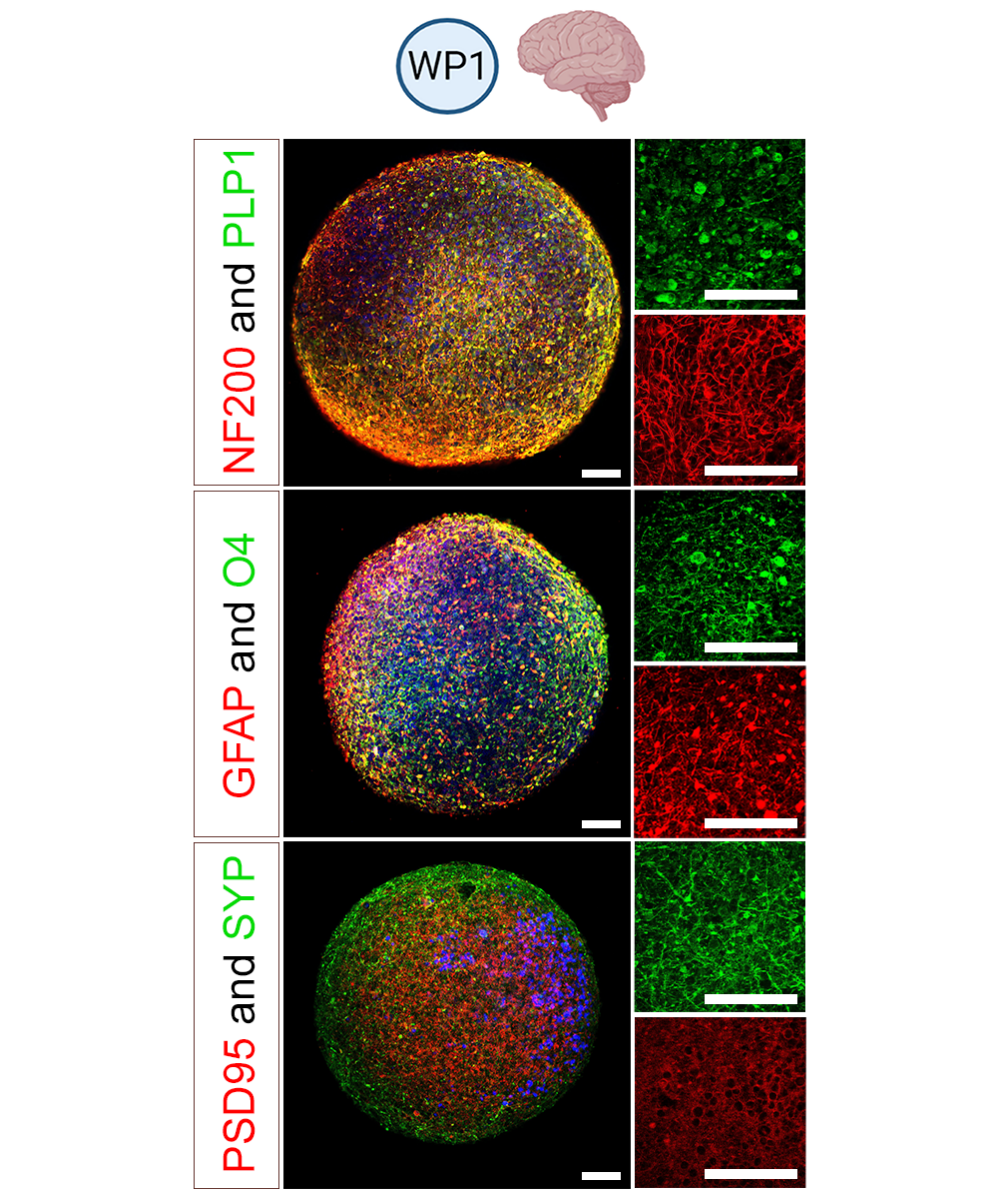
Brain model used in work package (WP) 1. Immunostainings of human induced pluripotent stem cell–derived 3D BrainSpheres after 8 weeks of differentiation, showing the presence of proteins specific for neurons (neurofilament heavy polypeptide [NF200]), synapses (postsynaptic density-95 protein [PSD95] and synaptophysin [SYP]), astrocytes (glial fibrillary acidic protein [GFAP]) and oligodendrocyte (proteolipid protein 1 [PLP1]). Scale bars: 40 µm.

Primary 3D hiPSC-derived brain cell cultures will be prepared and maintained as previously described [24]. This model contains neurons that form synapses, astrocytes, and oligodendrocytes myelinating the axons (Figure 3). Cytotoxicity will be determined by a resazurin assay after repeated exposure (7 d) to the selected glycol ethers (parent compounds and metabolites). Gene expression for cell type–specific genes, markers of synapses and myelin, and markers of cell stress will be quantified by quantitative reverse transcription polymerase chain reaction at concentrations of solvents under half-maximal effective concentration (EC50) for cytotoxicity. Immunostaining will be performed to assess the effects of solvents on synapses, myelin, and astrocyte reaction, and immunofluorescence will be quantified. NOAECs (Figure 2) will be determined for all tested endpoints, as previously shown for gene expression [42]. Brain cell cultures will also be exposed to the metabolites of PGME, propylene glycol butyl ether, and EGME and to the metabolites of the newly selected uncharacterized solvents, potentially produced by liver metabolism (WP3).

To establish the in vitro distribution kinetics of selected solvents necessary for toxicokinetic modeling, 3D brain cell cultures and medium will be collected 3, 6, 24, and 48 hours after the first exposure and after the last exposure of the repeated treatment. The solvent and its main metabolites (if relevant) will be quantified to establish a time course of disappearance from the medium and appearance in the cells as well as to assess the potential accumulation for the entire period of exposure. The fraction bound to culture plates’ plastic will be quantified after desorption. In silico modeling of glycol ethers in vitro distribution kinetics will then be developed. This model will be able to predict the change in cell-associated concentrations of solvents in BrainSpheres with time, as previously shown for amiodarone [43].

### WP2: In Vivo, In Vitro, and In Silico BBB Functionality Testing

**Figure 4:**
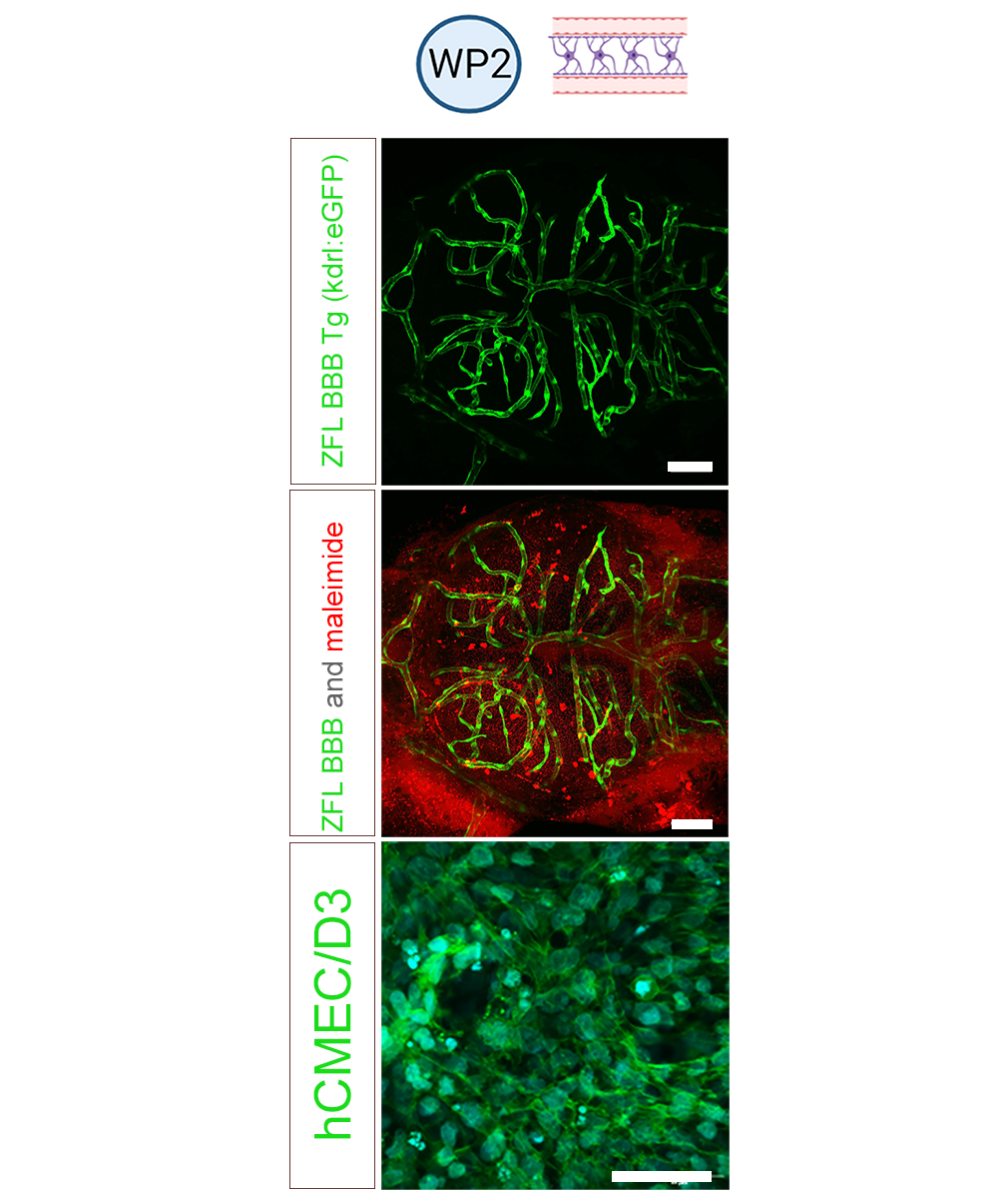
Blood-brain barrier (BBB) models used in work package (WP) 2: zebrafish larvae (ZFL) and human brain microvasculatur endothelial cells (hCMEC/D3). ZFL (2 top panels): tracer permeability across BBB. Dorsal view of the midbrain region of the zebrafish lines Tg (kdrl:enhanced green fluorescent protein [eGFP]), which expresses eGFP (green signal) in the endothelial cell membranes. ZFL were injected with the tracer 1 kDa maleimide (red signal). Scale bars: 50 µm. hCMEC/D3 cells (lowest panel): actin filament stained with fluorescein isothiocyanate phalloidin. Scale bars: 100 µm.

We previously established the zebrafish as a predictive vertebrate screening model to study the systemic circulation and tissue distribution of particulate drug carriers [44,45]. At 72 hours postfertilization, zebrafish embryos have a functional CNS and, presumably, a fully functional BBB. Anatomical structures such as the vascular endothelium can be visualized using transgenic fish lines expressing fluorescent proteins (Figure 4). Defined exposure of the zebrafish can be achieved by the simple addition of solvents to the fish incubation medium within a closed container. Other advantages of the model include the possibility of studying BBB functionality under physiological conditions in vivo and the high throughput. A well-known in vitro model for the human brain endothelium, hCMEC/D3 cell line (Figure 4) showing the formation of tight junctions and the expression of most transporters and receptors of the in vivo BBB [46], cultured in a transwell system, will also be used. Furthermore, extrapolation of in silico, in vitro, and zebrafish data to higher vertebrates seems feasible [47].

Zebrafish larvae are frequently used in developmental biology or toxicological studies. However, in this study, we will use zebrafish larvae exclusively to study BBB integrity and functionality [48]. Fluorescently labeled reference compounds (Figure 4) will be intravenously injected into the Duct of Cuvier, as markers of paracellular permeability (eg, fluorescein isothiocyanate dextran 70 or fluorescently labeled liposomes), substrates of drug export transporters (eg, rhodamine-123 as P-glycoprotein substrate), or nutrient transporters (eg, fluorescently labeled transferrin as a marker for receptor-mediated transcytosis). PGME will be the first reference compound to be tested because of its high water miscibility. To precisely assess exposure, analytical methods (gas chromatography-tandem mass spectrometry [GC-MS/MS]) will be used to determine the concentrations of solvents and their metabolites in zebrafish medium, in the headspace of closed incubation vessels and tissue samples (ie, zebrafish homogenates). Circulation, tissue distribution, and brain uptake of the reference compounds will be monitored by confocal laser scanning microscopy (live imaging of anesthetized fish embryos for up to 24 hours). The concentration-dependent toxicity of solvents or their metabolites will be monitored based on the viability and malformations of embryos. The integrity of vasculature will be visualized in transgenic zebrafish kdrl: enhanced green fluorescent protein embryos. The metabolic capacity of the zebrafish will be determined by quantifying potential metabolites (determined in WP3; Figure 2) in zebrafish tissue homogenates. The concentration-dependent toxicity to BBB and the coefficient of permeation of solvents will additionally be evaluated in the hCMEC/D3 cell line cultured in a transwell system.

Finally, quantitative estimates of passive cellular uptake and BBB permeability of solvents and their metabolites will be provided based on computational modeling using physicochemical molecular descriptors according to the methods we previously established [26,49]. These methods provide very high throughput, allowing the screening of web-based chemical libraries.

### WP3: In Vitro Hepatic Metabolism and Clearance

**Figure 5:**
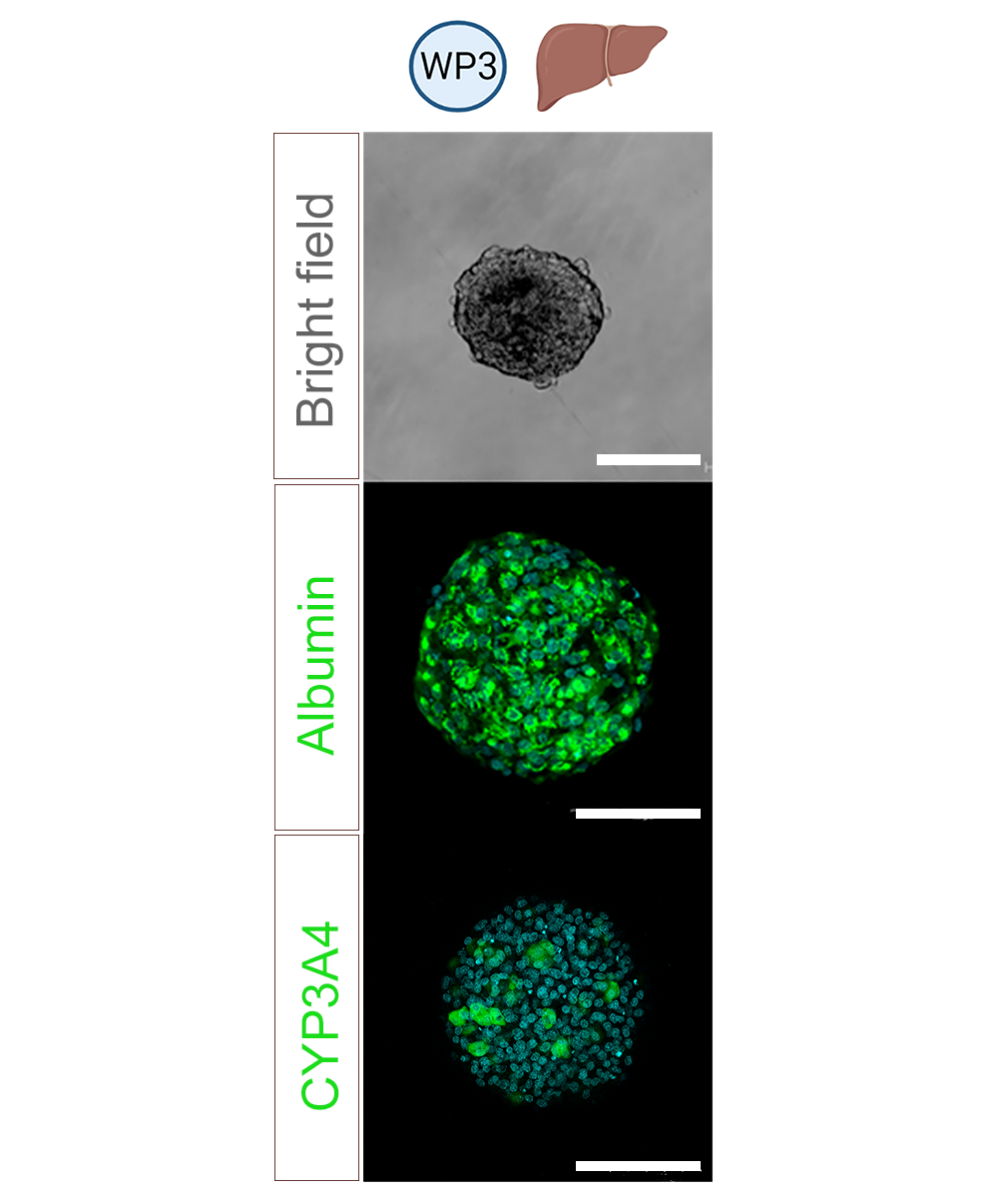
Liver model used in work package (WP) 3. Bright field and immunostainings of liver 3D HepaRG cultures showing the presence of albumin and cytochrome P450 3A4 (CYP3A4). Bars: 100 µm.

Because the liver is the main organ responsible for metabolism and a large contributor to compound clearance, we will implement a system suitable for predicting the hepatic metabolism of solvents. In recent years, 3D liver cell models have been proposed as an alternative to less physiological 2D cell monolayers, and their applications have progressed substantially [50]. They are widely used for the assessment of hepatotoxicity [51-55]. An advantage of spheroids is that they overcome the limitation of rapid decline of drug-metabolizing enzyme activities in primary human hepatocyte suspension culture and cell lysates [56], such as microsomes and liver S9 fractions. In this study, we will use 3D liver cultures of the well-characterized human HepaRG cell line (Figure 5), which represent a promising model to evaluate hepatotoxicity and hepatic metabolism [57].

Determining the appropriate experimental test system (eg, cell plate, incubation time, and exposure concentration) will be an essential part of the development of the 3D model. Moreover, analytical methods (GC-MS/MS and liquid chromatography-tandem mass spectrometry) to detect and quantify the solvents and the metabolites formed must be developed to calculate hepatic metabolism and clearance. Then, proof of metabolic competence and maintenance of the 3D HepaRG cells will be carried out by assessing the metabolism of known P450 substrates. The presence and secretion of albumin as a specific hepatocyte marker will be assessed using immunostaining and enzyme-linked immunosorbent assay. Solvent- and metabolite-induced cytotoxicity will be assessed after 48 hours and 7 days (repeated) of exposure to determine the nontoxic concentration range for subsequent experiments. The metabolic abilities of the 3D HepaRG model and 3D primary human hepatocytes will be compared. Furthermore, the clearance data obtained from the 3D HepaRG model will be compared with the short-term clearance measured in the human liver cell lysate (S9 fractions). In addition, Michaelis-Menten-Kinetic parameters (V_max_ and Km) for the formation of the metabolites will be derived using the S9 fractions. These data will be used to build a physiologically based toxicokinetic (PBTK) model (Figure 2).

### WP4: In Vivo Volunteer Exposure

Human biomonitoring refers to monitoring exposure-related health risks by analyzing biological samples, usually blood and urine samples [58]. The biomonitoring limit values (BMLVs) are set to protect human populations against the potential toxic effects of chemical substances. These limit values account for all routes through which a chemical can enter the body. These are most often the inhalation and skin routes in occupational and environmental settings. Kinetic studies that provide absorption, biotransformation, and elimination rates as well as the absorption and elimination half-lives of the parent compound and its metabolites are necessary to set BMLVs. The apparent urinary elimination half-lives of the parent compound and its metabolites will later be used to develop a biomonitoring method. Sample collection time is crucial and is determined by the apparent elimination half-life of the chemical. Blood concentrations will be used to calibrate the air:blood PC for the toxicokinetic models.

We will recruit 4 participants for 2 of the selected solvents. All participants must meet the following criteria: they should be healthy individuals who do not smoke or use contraceptive hormones, do not consume alcohol, be aged between 18 and 65 years, have normal red blood cells and hemoglobin concentrations, maintain a BMI between 18 and 25, and should not be working with glycol ethers. Pregnant and breastfeeding women will be excluded from this study. Participants will be recruited using flyers and announcements distributed at the teaching hospital, university websites, and bulletin boards. All participants will sign a written informed consent form before being included in the study.

The participants will be exposed to a single glycol ether for 4 hours under controlled conditions in an exposure chamber (12 m^3^). PGE concentrations will be set at or below the Swiss occupational exposure level (OEL) if one exists. In the absence of an OEL, we will rely on existing OELs for other propylene glycols. The parent compound (free and conjugated) and the oxidative metabolites (free and conjugated) of the selected glycol ethers will be monitored in blood, urine, and exhaled air samples. These are noninvasive methods used for human participants, and the results will be used in WP5 to estimate brain concentrations. All compounds will be quantified using capillary gas (parent compound in blood, urine, and exhaled air) or liquid (metabolites in blood and urine) chromatograms with tandem mass spectroscopy detection.

### WP5: In Silico PBTK Modeling

PBTK models can be used to estimate human brain concentrations. The risk of neurotoxic effects can be estimated by comparing the predicted solvent-brain concentrations with the NOAEC obtained from the in vitro models. Mathematical models such as PBTK models can be used to predict the ADME of a chemical and its metabolites. In these PBTK models, the body is represented by 1 or more compartments. Each compartment represents 1 or more tissues that are kinetically homogeneous, that is, that have similar perfusion rates and an assumed similar substance solubility. PBTK models are described by a set of parameters that define the compartments and a set of mass balance differential equations for each compartment.

A previously developed toxicokinetic model for PGME with metabolism that is assumed to follow Michaelis-Menten kinetics calibrated for different age groups serves as the basis for our development [30,31,59]. We aim to modify this previously developed toxicokinetic model to include a separate compartment for the brain using BBB flux rates obtained from in vivo, in vitro, and in silico models (WP2). In addition, we will implement PC obtained from empirical human experiments (WP4) and metabolic parameters assessed in a hepatocyte assay (WP3). The toxicokinetic models will be able to simulate not only acute but also chronic exposures; therefore, both short-term and long-term exposures can be explored in silico. We will develop the toxicokinetic model into a physiologically based pharmacokinetic model based on the existing inhalation-only toxicokinetic model originally developed for PGME [40] and build it in the Berkeley Madonna software or equivalent. We will model the brain as a single compartment with direct contact with the blood flow and where organic solvent uptake will be assumed to be diffusion limited, which is in line with other physiologically based pharmacokinetic models [60]. Values for physiological parameters (volume of vascular brain, as fraction of brain volume [FVvb], volume of extravascular brain, as fraction of brain volume [FVevb], volume fraction of brain tissue [FVB; as percent of body weight], BBB surface [Sh] in cm^2^, fraction of cardiac output in brain at rest [BFbrainrest]/cardiac output in brain at light work [BFbrain]) required to build the TK model are from the scientific literature. Depending on the substance, values of chemical-specific parameters such as the pulmonary retention (Rpulm), central:air PC (Pca), blood:air PC (Pba), and brain tissue:vascular brain PC (Pevb_vb) are either taken from the literature or estimated in silico. Since the partitioning of organic compounds between human tissue homogenate and blood is a function of water and lipid content of tissues and the n-octanol:water PC (Kow), PCs are estimated in silico based on LogKow. Kinetic coefficients needed for each organic solvent included in this study will be found in WP3 for liver metabolism (Michaelis-Menten parameters [V_max_ and Km]), WP2 for BBB uptake (BBB permeability-surface area product [PS]). The fraction unbound in blood (Fu_blood) will be estimated based on the fraction unbound in plasma (Fu_plasma) and the blood-to-plasma ratio (Rb), and the fraction unbound in brain (Fu_brain) will be considered when modeling each solvent as only the free fraction is able to distribute to different tissues and is biologically active. The model will be calibrated by comparing the predicted and actual urinary organic solvent concentrations obtained from the controlled human experiments (WP4). Both the free and total organic solvent concentrations (free+conjugated) will be obtained for calibration.

## Results

With this project, we expect to provide a strategy to rank uncharacterized solvents and their potential liver-formed metabolites, according to their potential neurotoxicity, and in comparison with the banned EGME. More importantly, a series of PBTK simulations will be conducted to predict occupational exposure, assuming 8 hours of exposure per day, 5 days per week, physical activity for 12 hours per day, and rest for the remaining 12 hours. We will use the PBTK model in reverse dosimetry to estimate air concentrations that do not produce brain concentrations determined as neurotoxic in the hiPSC-derived 3D brain model. We will recommend that authorities setting occupational exposure and public health limits consult these values. Keeping the exposure below the brain effect level should ultimately increase the protection of exposed workers and the general population with domestic exposures.

We also anticipate gaining insights into the mechanisms of action of solvents of the glycol ether family. We will elucidate the possible toxic endpoints in the brain, liver, and zebrafish models. Furthermore, we will be able to establish how toxicity is related to the compounds’ lipophilicity and metabolites.

## Discussion

Overall, our strategy combining multiple, fit-for-purpose 3D advanced cell culture systems; zebrafish larvae; biomarker analysis; human ADME experiments; and in silico prediction is expected to contribute to the improvement of human risk assessment. Although we identified some risks we could encounter during the project, we are confident that our already determined mitigation measures will be able to overcome potential pitfalls.

Determination of the passage of solvent through the BBB may be challenging; hence, we are applying 3 different complementary methods: in vivo zebrafish larvae, in vitro human cells (hCMEC/D3), and in silico models. We are also considering and assessing the effects of the hepatic metabolites of the solvents on human BBB cells. With this experimental strategy, issues regarding the potential direct effect of solvents on cell membranes, the relatively low miscibility of solvents with water, and the physiological differences between zebrafish and humans (eg, metabolism and route of expected) should be overcome.

We have extensive experience in recruiting human volunteers for controlled human exposure sessions in the exposure chamber. Sometimes, recruitment takes longer than anticipated, and if that is the case, we will extend the timeline to not compromise the size of the study. New analytical chemical methods will need to be determined, which is time consuming. However, we will use a laboratory with extensive experience in analyzing PGME in urine and blood samples. This will also have to be accommodated with a delay in the timeline.

Future developments, not included in this study, are a strategy extended to include developmental neurotoxicity by determining other endpoints, such as proliferation and neurite outgrowth, after exposure of BrainSpheres to solvents at earlier developmental stages and by adding an in vitro test system to take into account the passage of solvents through the placental barrier [61]. We might also consider combining zebrafish embryo behavioral assays (eg, spontaneous tail coiling) with the BrainSpheres model as readouts for developmental neurotoxicity. Finally, the PBTK model could be adapted to determine solvent air concentrations that are unlikely to cause neurotoxic effects in fetuses or pregnant women.

## Abbreviations

ADME: absorption, distribution, metabolism, and excretion
BBB: blood-brain barrier
BFbrain: cardiac output in brain at light work
BFbrainrest: cardiac output in brain at rest
BMLV: biomonitoring limit value
CNS: central nervous system
EGE: ethylene glycol ether
EGME: ethylene glycol methyl ether
Fu_blood: fraction unbound in blood
Fu_brain: fraction unbound in brain
Fu_plasma: fraction unbound in plasma
FVB: volume fraction of brain tissue
FVevb: volume of extravascular brain, as fraction of brain volume
FVvb: volume of vascular brain, as fraction of brain volume
GC-MS/MS: gas chromatography-tandem mass spectrometry
hCMEC/D3: human brain microvascular endothelial cells
hiPSC: human induced pluripotent stem cell
NOAEC: no observed adverse effect concentration
OECD: Organisation for Economic Co-operation and Development
OEL: occupational exposure level
Pba: blood:air partition coefficient
PBTK: physiologically based toxicokinetic
PC: partition coefficient
Pca: central:air partition coefficient
Pevb_vb: brain tissue:vascular brain partition coefficient
PGE: propylene glycol ether
PGME: propylene glycol methyl ether
PS: blood-brain barrier permeability-surface area product
Rb: blood-to-plasma ratio
Rpulm: pulmonary retention
Sh: blood-brain barrier surface
TG: Test Guideline
Vmax: maximum velocity
WP: work package

## Data Availability

Data will be available from the corresponding author on reasonable request.
